# Minor Injuries, etc.

**Published:** 1887-03-05

**Authors:** 


### Till the Doctor Comes.

[Inquiries and Communications for this column should be addressed, "Physician," care of the Editor of THE HOSPITAL.]

### XII.—MINOR INJURIES AND AILMENTS.

*First help in cases of Accident.*—Carry the patient as gently as possible to the nearest and most convenient place. Carefully guard all wounded parts, and protect them from further injury. Note if there are any wounds, or much bleeding, taking such steps to stop the latter as have been indicated. A shutter or hurdle is much more pleasant than a jolting cart for his removal, but the men must take care to keep in step. If there is much collapse, give some brandy and water, but not in the reckless manner that is usual. Loosen all the clothes about the neck, especially the collar and necktie. Having reached home, or the place where the patient is to remain, warm a bed and prepare to get him into it quickly. If the feet are very cold, put hot bottles, or some hot bricks wrapped in flannel, against them. Remove the clothes with the least disturbance possible, especially from any injured part; do not destroy them unnecessarily, but where practicable cut up the seams. The boot and stocking in an injury to the foot must usually be sacrificed, and the outer trousers-seam slit. If the arm is injured, remove the clothes from the uninjured arm first; it will then be easy to slip them off the other. In the case of an injury to the head, keep it raised on pillows, and put cold rags to it. It is the rule in hospitals for every accident (except in special cases) to be thoroughly washed all over, or at any rate the limbs, before being put to bed, and this rule may well be applied to all cases. Having thus made the patient comfortable, as far as you know how, prepare for the doctor's visit by getting ready for him anything he may require—hot water, rags, basins, jugs, scissors, lint, etc.; much valuable time will often be thus saved.

*Bruises.*—These require in the first place rest—without which no wounded part can do well. At the discretion of the doctor, or according to the inclination of the patient, bruises may be treated at first with either hot fomentations (see p. 357) or cold evaporating lotions (see p. 357). If on the body, the former must almost necessarily be applied, but if on the limbs, the latter will often be more serviceable. When the inflammation has subsided, and the discolouration is beginning to fade, some stimulating liniment will help to hasten this stage, and equal parts of soap and compound camphor liniments will be found very agreeable, or the old remedy of hartshorn and sweet oil mixed in the proportions of one part of hartshorn, one of water, and two of sweet oil. When beginning to use the injured part, the support of a bandage skilfully applied will be found most serviceable.

*Sprains.*—These require much the same treatment as bruises if treated by an amateur. Some persons recommend leeches in bad cases; but they should not be used except by the orders of a surgeon. After the first week, strapping—firmly and evenly applied to the whole of the uninjured joint—will afford great support and much ease. When the joint is again to be used, the above-mentioned liniments rubbed into the part and a firm bandage afterwards applied will give strength and support to it. Sir Erasmus Wilson recommends that in these cases some warm lard should be taken and rubbed into the sprained part for half or three-quarters of an hour; some cotton-wool must then be wrapped round the joint, and a light bandage applied. He states that sprains thus treated recover much more rapidly than others.

